# Digital Tools for Decision Support in Social Rehabilitation

**DOI:** 10.3390/jpm15100468

**Published:** 2025-10-01

**Authors:** Valeriya Gribova, Elena Shalfeeva

**Affiliations:** Institute of Automation and Control Processes Far Eastern Branch of the Russian Academy of Sciences, 5, Radio St., Vladivostok 690041, Russia; gribova@iacp.dvo.ru

**Keywords:** social rehabilitation, level of rehabilitation potential, rehabilitation measures, knowledge base, semantic model

## Abstract

**Objectives**: The process of social rehabilitation involves several stages, from assessing an individual’s condition and determining their potential for rehabilitation to implementing a personalized plan with continuous monitoring of progress. Advances in information technology, including artificial intelligence, enable the use of software-assisted solutions for objective assessments and personalized rehabilitation strategies. The research aims to present interconnected semantic models that represent expandable knowledge in the field of rehabilitation, as well as an integrated framework and methodology for constructing virtual assistants and personalized decision support systems based on these models. **Materials and Methods**: The knowledge and data accumulated in these areas require special tools for their representation, access, and use. To develop a set of models that form the basis of decision support systems in rehabilitation, it is necessary to (1) analyze the domain, identify concepts and group them by type, and establish a set of resources that should contain knowledge for intellectual support; (2) create a set of semantic models to represent knowledge for the rehabilitation of patients. The ontological approach, combined with the cloud cover of the IACPaaS platform, has been proposed. **Results**: This paper presents a suite of semantic models and a methodology for implementing decision support systems capable of expanding rehabilitation knowledge through updated regulatory frameworks and empirical data. **Conclusions**: The potential advantage of such systems is the combination of the most relevant knowledge with a high degree of personalization in rehabilitation planning.

## 1. Introduction

In contemporary society, the integration of individuals with impaired socio-psychological adaptation into community life has become a critical aspect of social policy. Assistive technologies in rehabilitation not only compensate for lost social resources but also mitigate potential risks of maladjustment. Current trends emphasize the need for personalized and timely rehabilitation approaches.

Social rehabilitation is a systematic process aimed at restoring lost social connections and functions for individuals requiring reintegration. Target populations include those with

• Addictive disorders (substance abuse, alcoholism, gambling);
• Mental health conditions (schizophrenia, anxiety disorders, personality disorders);
• Comorbid biopsycho-socio-spiritual maladjustments linked to non-medical psychoactive substance use.

The implementation of social rehabilitation relies on the following core principles:

1. Timeliness: Early identification of issues and structured intervention.
2. Comprehensiveness: Holistic support systems integrating multiple interventions.
3. Individualization: Tailored rehabilitation strategies based on specific needs.
4. Accessibility: Equitable availability of services for all beneficiaries.

These principles align with global rehabilitation standards, including patient-centered clinical guidelines aimed at improving outcomes [1].

Modern practice involves multidisciplinary teams in clinical decision-making, increasing accountability for therapeutic consequences. A salient example is irrational pharmacotherapy in psychiatry, where inappropriate psychotropic drug use exacerbates symptoms, reduces remission quality/duration, and can contribute to the transition of the disorder to a persistent, long-term condition. [2].

Despite inherent challenges in formalizing this domain [3,4], advances in evidence-based medicine and digital technologies now enable knowledge-driven decision support systems (DSS) [2].

In this context, the following elements are of particular importance:

1. Regulatory frameworks for clinical decision-making;
2. Mandatory consideration of individual clinical characteristics;
3. Systematization of professional expertise, including independent peer review mechanisms (“third opinion”).

Current research in artificial intelligence focuses on developing and validating innovative models and methodologies aimed at creating intelligent systems to support rehabilitation processes. Particular emphasis is placed on designing algorithms that enable personalized rehabilitation approaches for patients. These systems are built upon expert knowledge that formalizes the professional experience of practicing specialists.

The implementation of such intelligent solutions is driven by two key factors:

1. The need to enhance the quality of medical decision-making
2. The requirement for objective quality control in healthcare delivery

These intelligent systems are particularly valuable in medical domains where the consequences of erroneous decisions can be severe. Their application not only helps minimize medical errors but also substantially improves the overall quality of care provided.

However, the existing software has limitations in comprehensively supporting rehabilitation activities [5].

Various intelligent software solutions for social rehabilitation include

− Telepsychology platforms offering online consultations with psychologists;
− Mood-tracking applications for emotional state monitoring;
− Group therapy and support services connecting individuals with similar challenges;
− Interactive training programs for social skills development;
− AI-powered assistants for monitoring and managing conditions of people with disabilities.

These tools assist in supporting and restoring social functioning, helping achieve certain levels of social, material, and spiritual independence.

Semantic models and methods have proven effective for representing complex knowledge and simulating human reasoning processes. They incorporate rules for structured information organization; methods for information interpretation; logical inference mechanisms [3,4].

The research aims to present interconnected semantic models that represent expandable knowledge in the field of rehabilitation as well as an integrated framework and methodology for constructing virtual assistants and personalized decision support systems based on these models.

As the proposed semantic models as the ontologies of González et al. [4] are based on the classical (1993) definition of Gruber: “a specification of a conceptualization”. Contrary to the use of an ontology for the integration of applications [4], we use a single ontology (semantic models complex) of the medical portal for the consistency of the parts within the DSS, and one of its parts—the semantic model of electronic health records—for the integration with third-party applications. Such integration is possible because (a) its structure contains all the typically documented information about the patient, (b) the formalization of the patient document allows conversion into Structured Electronic Medical Records (SEMRs) of other organizations or medical systems. The dictionary of clinical terms with references to identifiers of other dictionaries is used for writing documents.

## 2. Materials and Methods

### 2.1. The Materials

To achieve the research goals, it was necessary to analyze the knowledge used in decision-making for rehabilitation.

The social rehabilitation process involves the following stages:

1. **Diagnostic and Prognostic Stage**

Specialists assess the individual’s condition and conduct an expert evaluation to determine their potential for rehabilitation. Based on the findings, they develop a personalized rehabilitation plan based on the findings.

2. **Planning Stage**

Optimal forms, methods, techniques, and technologies are selected to implement in the specific case.

3. **Implementation Stage**

The comprehensive rehabilitation program is carried out, with ongoing monitoring of the individual’s progress and necessary adjustments made as required.

4. **Final Stage**

Data collected during monitoring is systematized, and the outcomes of the rehabilitation efforts are evaluated.

#### 2.1.1. Diagnostic and Prognostic Stage

For diagnostic purposes, it is essential to understand both typical and rare manifestations of biological, psychological, social, and spiritual disorders and their different stages, as well as the factors that influence symptom presentation or the evolution of the clinical picture. A hallmark of mental illness is its prolonged course, which often persists for years or even a patient’s entire lifetime. Most psychiatric disorders are characterized by alternating periods of exacerbation and temporary remission.

The diagnostic process involves multiple procedures: anamnestic evaluation by a specialist, clinical psychotherapeutic interviews with a clinical psychologist (life history assessment), comprehensive examinations by a therapist, neurologist, and other specialists [6].

Clinical practitioners routinely use standardized scales to assess the current status and potential for rehabilitation as well as a thorough analysis of the medical history and symptoms reported by the patient. They also use specialized examination and interview techniques to evaluate alcohol-use motivation. Psychologists or occupational therapists conduct special assessments using structured questionnaires [7].

In the field of psychiatry and addiction treatment, a scale called the *level of rehabilitation potential* (LRP) is used to assess the potential for rehabilitation of drug and alcohol addicts. The scale consists of several blocks of questions, including premorbid characteristics, clinical features of the addiction, social status, and social consequences. It also includes questions about changes in personality that have occurred as a result of the addiction. Experience with the scale has allowed researchers to develop methods for identifying the degree of anxiety and anger among patients using different drugs. For example, those using opium-based drugs tend to have the highest levels of anxiety. Additionally, the scale can help identify affective disorders such as depression, anxiety, panic attacks, social phobias, eating disorders, and personality disorders. To determine the severity of these affective disorders, the Beck Depression Inventory is often used. This inventory includes a list of common symptoms of depression and allows clinicians to assess the severity of each symptom in a patient.

The use of standardized methods in drug addiction practice allows specialists to quantitatively assess the severity of pathological manifestations. By using valid assessment scales and tests, it is possible to find statistically reliable relationships between measured values and ensure the receipt of reproducible research results and scientifically based practical recommendations [8].

In order to increase the objectivity of the analysis, researchers can correlate the identified clinical signs with codes of impairments and limitations of life activities in accordance with the International Classification of Functioning, Disability, and Health (ICF). This classification system, based on the biopsychosocial model, provides a standardized assessment of various aspects of health, including body functions and structures; activity and participation level; and influence of environmental factors. The main goal of the ICF is to integrate data on the state of health and the dynamics of the disease, taking into account biological, social, and personal aspects. A rehabilitation diagnosis is a detailed description of the impairments of the structures and functions of organs and systems that arose in a patient after a stroke. These impairments lead to limitations in activity and participation, and environmental factors can both facilitate and hinder the patient’s performance of everyday tasks and functions [9].

In the course of professional practice, specialists identify consistent correlations between

1. Observable behavioral manifestations;
2. Severity of pathological alterations;
3. Compensatory adaptation mechanisms;
4. Clinical diagnosis;
5. Feasible rehabilitation objectives [9].

For patients with alcohol dependence, key therapeutic goals comprise

• Establishing sustained remission (abstinence);
• Restoring social functioning;
• Rehabilitating vocational competencies;
• Normalizing personal status.

*For prognostic activities*, it is important to know and accumulate patterns about the relationship between a set of disorders and identified factors with possible goals and the degree of their achievability. Rehabilitation potential is understood as a set of several patient abilities: physical; mental; spiritual; social. Patients can be assessed to have “high LRP”, “average LRP”, or “low LRP” based on a prognostic assessment of the patient’s abilities during long-term remissions, the patient’s further adaptation, and the patient’s return to normal social life. The LRP assessment scale consists of several blocks of questions. The scores are used to assess the patient’s physical and mental condition, social development, and disease severity. The scale is based on objective data on heredity, pre-morbidity, somatic condition, severity, and consequences of drug addiction, features of personal development, and social status of patients. The answer to each question has a quantitative expression from +5 to -3 points. Depending on the total numerical score, three levels of rehabilitation potential are distinguished: high (83 points (±6) with an “ideal” of 110), average (62 points (±10)), and low (45 points (±10)). Based on the evaluation received, the observing specialists obtain a complete picture of the patient’s characteristics, emotional and volitional state, and moral sphere. Alcoholics with a high potential for rehabilitation receive an average of 150 points (±6), the “ideal” score being 187 points, while those with average and low potential receive an average score of 61 (±10) and 10 points (±10), respectively.

#### 2.1.2. Planning Stage

When planning rehabilitation measures, it is important to know the range of methods for each problem, factors of their effectiveness, and complex effects (when it is necessary to work not only with the patient, but also with his relatives). Based on the results of tests, psychological diagnostics and analysis, specialists develop an individual treatment plan for a specific patient.

Planning complex rehabilitation depends on the goals and diagnosis established in detail. Personalization of the rehabilitation process is requires taking into account the individual characteristics of the patient, which ensures the most effective recovery [10]. Personalized programs for restoration of lost connections and functions include formulation of a diagnosis, setting of goals, and planning of interventions, paying attention to improvement in interaction between the patient and the therapist [10,11].

The choice of the method of complex rehabilitation is made taking into account the results of clinical and psychological examination and the level of rehabilitation potential [12].

For patients with alcohol dependence syndrome, who have lost their social and psychological adaptation and who have lost or significantly reduced their level of work qualifications, the use of occupational therapy is typical. Determination of working conditions based on the LRP allows us to assess the complex medical, psychological, and social factors that determine the specificity of the clinical picture of a given patient and the possibility of restoring impaired social functions, which will create conditions for participation in work activities and the formation of long-term high-quality remissions. The use of standardized methods in drug treatment practice allows us to choose treatment tactics depending on the structure and severity of existing disorders [8].

Therapeutic methods can also be aimed at improving the patient’s socio-psychological adaptation. Refusal of psychoactive substances can be considered as a result of a thorough analysis of the patient’s personality and system of values. Symptomatic treatment is suggested: elimination of insomnia, pathological influence on psychoactive substances, and selection of long-term psychopharmacotherapy [6]. A common means of increasing the reserve capacity of the body is the introduction of regular exercise therapy.

Knowledge about the goals of rehabilitation and methods for their achievement is contained in regulatory documents, clinical guidelines, and experience accumulated, and systematized through collections of precedents, datasets, and their processing. In such sources of knowledge, sets of objective indicators and observations are associated with important goals, prognosis, and rehabilitation program, etc. For example, a clinical psychologist will link certain values of ICF codes b1400 (attention span), b1561 (visual perception), and d160 (concentration of attention) with the goal of “increasing criticism of one’s condition”.

Examples of linking a set of objective indicators (“Montreal Cognitive Assessment Scale”) and observations from the “Neuropsychological Examination” block to the prognosis and rehabilitation program are as follows. The memory restoration program includes “memorizing visual images,” “memorizing pairs of words related in meaning (associative memory),” and “memorizing stories” corresponding to the condition of a patient with severe impairments of modality-specific operational and auditory-verbal memory and mild impairments of short-term and long-term memory and 0 points for the “memory” item on the Montreal Cognitive Assessment Scale.

Clinical psychologist will associate certain values of ICF codes d160 (attention) and b1140 (time orientation) or impairments found during examination and testing (mild impairments in thinking: Visual-Figurative and Verbal-Logical; and 4 points for “Time Orientation” on the Mini-Mental State Examination (MMSE) and 3 points for Visual-Constructive or Executive Skills on the Montreal Cognitive Assessment with the program for restoring constructive activity, including copying objects and geometric figures, completing the drawing of objects, constructing various parts, and with the program for restoring representations of the body scheme “assembling a human face from parts”.

If the assessment of attention shows significant impairment of concentration and voluntary attention, and mild impairment of switching and separation, and the item of attention (4 questions) receives 0 points (in the Montreal Cognitive Assessment Scale), the prognosis of agnosia is favorable, and it is useful to include in the rehabilitation plan the program of restoration of attentional functions for the stage of moderate disorders which include performing exercises that require increased concentration and simultaneous retention of several instructions: Schulte-Gorbov tables, proofreading tests (with two or more given letters), mazes, searching for differences, missing details.

Sometimes it is important to solve the “addiction problem”. It is more serious than just the physical discomfort of stopping use, because it has a psychological component. This includes

− psychological personality traits that once led to the onset of use;
− a false attitude that all problems and difficulties are “easily solved” by use;
− obsessive thoughts, memories of using;
− dreams associated with using;

and so on.

#### 2.1.3. Implementation Stage and Final Stage

Several criteria are used to monitor ongoing changes and evaluate the results. The evaluation of the appropriateness of a physician’s treatment regimen and the patient’s response to treatment is inseparable from the concept of quality of psychiatric care. There are several tools available for assessing the effectiveness of treatment for mental health conditions: (1) physician’s assessment—this can be influenced by a physician’s biases; (2) patient’s self-report—this is free from some of the limitations of a specialist’s assessment but may not be suitable for patients with cognitive impairment or low motivation; (3) other tools, such as standardized scales or questionnaires, which can help objectify the data collected. To avoid subjectivity in assessing a doctor’s decision, it is recommended to follow the recommendations of psychiatrists who focus on the remission or recovery of the patient as an indicator of treatment effectiveness [13].

Psychiatric patients who are frequently or extremely frequently rehospitalized are a group for which specialized care is ineffective, and the level of rehospitalization remains one of the main criteria for the quality of psychiatric care. Reducing the risk of rehospitalization is largely achieved by appropriate methods and treatment regimens chosen by doctors. The automatic assessment of the quality of psychiatric care in a hospital consists of determining the difference between the date of discharge of a given patient and the date of his next hospitalization. Thus, in the case of patients with diseases such as “schizophrenia” and “anxiety disorders”, the treatment during the previous hospitalization should be recognized as inadequate if the time indicators of the stable remission phase achieved then are low (up to half a year), which indicates insufficient measures taken by the doctor [2].

### 2.2. The Methods

The knowledge and data collected in these areas require specialized tools for their presentation, access, and utilization. To create a set of models that serve as the foundation for decision support systems in the field of rehabilitation, it is essential to

1. Conduct a thorough analysis of the domain, identifying concepts and categorizing them into relevant groups, as well as establishing a comprehensive resource that encompasses the necessary knowledge for intelligent support;
2. Develop a series of semantic models that accurately represent the knowledge required for the rehabilitation of patients.

Creating a collection of ontological models represents one of the most challenging and crucial stages in designing decision support systems, demanding significant intellectual effort and responsibility.

Semantic models and ontologies are not intended to replace human understanding; rather, they are designed to structure the information used in discussions and decision-making processes. Our focus is on aspects that can be operationalized in clinical practice. Our ontology is the result of identifying key “clinically significant” elements and their connections. It formalizes the relationships of key components and target indicators that are already established in clinical practices. Thus, it ensures machine comprehensibility of knowledge graphs created according to the ontology. Identifying “clinically significant connections between concepts” is necessary for integrating data and knowledge and supporting decision-making in the context of rehabilitation.

The development of the complex of semantic models (ontologies) was carried out on the IACPaaS cloud platform (https://iacpaas.dvo.ru, accessed on 31 January 2025), which implements a two-level approach for the representation and formation of data and knowledge. The first level defines the structure and rules of knowledge/data formation, the second level defines the knowledge/data formed according to the structure and rules [14].

The composition of the set of ontological models sufficient for forming components of decision support systems for patient rehabilitation is determined by the following factors:

(1) Structure all information on patient management tactics, including templates for examination by a multidisciplinary rehabilitation team (MDRT),
(2) Formalize the key elements and characteristics of the research methods used, as well as their connections with the decisions made regarding the goals and means of rehabilitation.

Accordingly, it should be possible to support the entry of such information about the patient, save it in the form of information resources and generate accounting documents.

To ensure these processes, reference books and terminological databases are needed, including a dictionary for collecting patient anamnesis and complaints, examining specialists, objective observations, and forming conclusions. Resources for entering clinical data should contain descriptions of scales and methods of examination, examination, and interview of patients. Resources for documenting and storing clinical data should contain the patient’s medical rehabilitation record or medical history.

To minimize the risk of oversimplification, our ontology (and methodology) was developed and validated in close cooperation with doctors, including ongoing interaction with clinical experts. The «criterion of truth» for our model is the practical usefulness of the knowledge formalized according to our ontology: Do the DSS with this knowledge increase the consistency of work of the multidisciplinary team? Do they improve the decision-making process? Two methods are used to evaluate this: technical analysis of the advice generated, and analysis of feedback from doctors.

Two principles are often used to deduce the optimal solution: logical inference based on formalized knowledge and case-based reasoning (precedent-based inference). Due to the specifics of medicine, cases are accumulated and used in both the search for solutions and the evaluation of decisions made. The characteristics of the case base continuously improve over time with the accumulation of experience [2].

The choice and application of the IACPaaS cloud platform for the creation of DSS provides an opportunity for independent participation of different groups of specialists from both practical and scientific levels of healthcare. Experts, engineers, and researchers are involved in creating these types of resources. The approach ensures the linking, consistency, and reuse of ontological components [14].

To ensure reliability and practical value, we have implemented a multi-level quality assurance system with verification procedures.

(1) We use reliable and trustworthy sources, such as national clinical guidelines and standards, to form the knowledge base. The final decision on their selection rests on the expert.
(2) The IACPaaS platform for ontological developments allows us to check the syntactic and partial semantic correctness of formalized knowledge and highlight contradictions for resolution.
(3) Before transferring to experimental operation, validation is performed on collections of completed real case histories selected by experts.
(4) IACPaaS decision support systems are of the Explainable AI type. The principle of explainability is implemented so any advice or conclusion is accompanied by an explanation, which is generated based on the knowledge base. This allows the doctor to critically evaluate the reasonableness of the recommendation and make a balanced decision.

Thus, our guarantee of veracity is based not on blind trust in algorithms, but on several levels of control with the mandatory participation of a human expert in key stages; on transparency and explainability of any system action; on continuous validation through real-world case studies.

## 3. Results

A systemic analysis of the patient’s rehabilitation process was carried out in collaboration with healthcare professionals, and the information collected, analyzed, and documented was formalized and structured. Semantic models were constructed. We focus on aspects that can already be operationalized in clinical practice.

**Figure 1:**
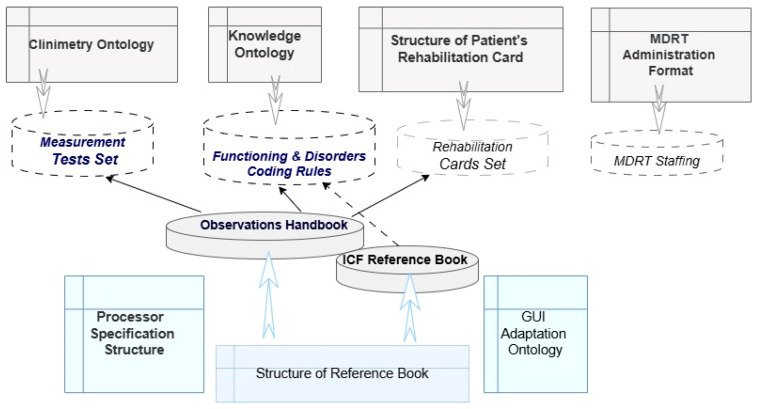
Scheme of semantic model complex.

For example, standardized “tools” such as tests and surveys with rating scales help to translate the subjective assessment of a person’s state into more formalized scores. This technique is reflected in the Clinimetry Ontology (Figure 1).

The knowledge ontology (Figure 1) records the fact of the connection of these scores with other factors, with a functional diagnosis, with treatment measures, etc. It establishes important relationships between professional concepts described in the medical literature. In particular, it provides a “language” for directly determining rehabilitation potential through a single rating scale LRP or through a set of condition ratings, a set of observations, and factors from the medical history. Knowledge of ICF diagnostics is formed as links between each ICF code for a specific disorder and a set of specific observation values. Accumulating knowledge about the rules for coding physical disorders and making rehabilitation diagnoses is an important stage in the MDRT’s work [9].

The *Structure of Patient’s Rehabilitation Card* (Figure 1) has been defined. It enables clinical specialists to record all necessary medical information about the patient and reflects the evolution of the recorded data.

Based on the constructed semantic models, a knowledge base (rules for coding functioning and disorders), Measurement Tests Set and repository of Rehabilitation Cards were formed on the IACPaaS cloud platform (Figure 1). The constructed semantic models are also the basis for the logic of reasoning based on knowledge (for intellectual support in the rehabilitation of patients).

**Figure 2:**
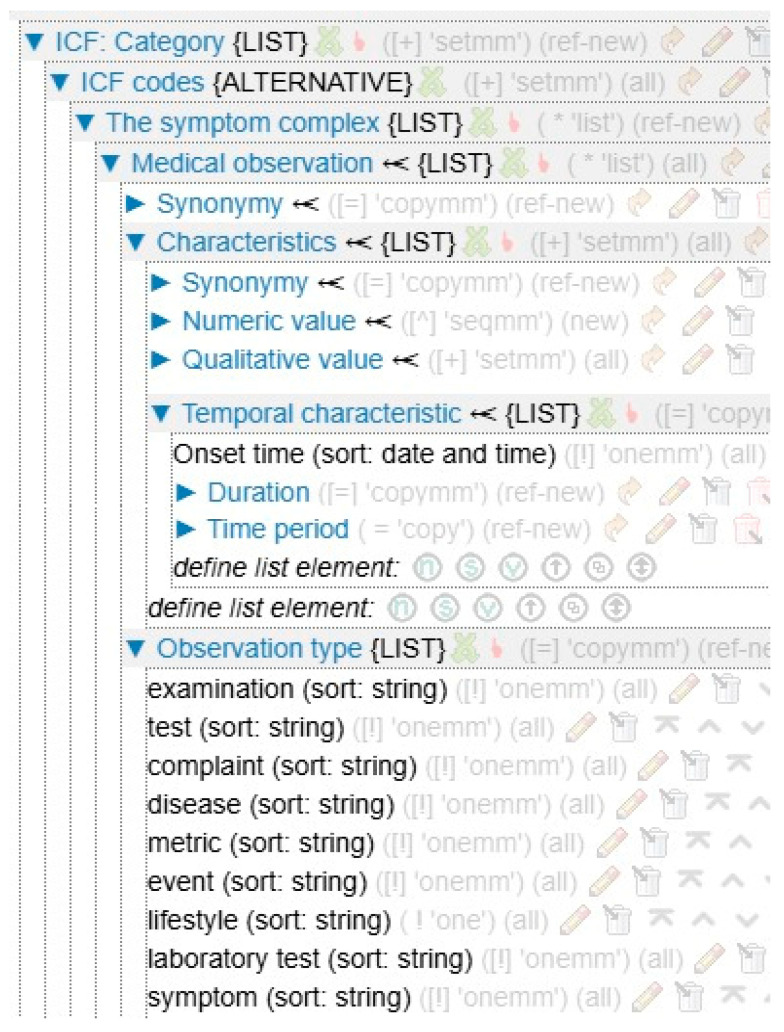
Fragment of the ICF-diagnostics semantic model.

Semantic models of diagnostic knowledge according to ICF (Figure 2) reflect the structure of knowledge about the presence of disorders (according to domains B, D, S, E—body functions, body structures, activity and participation, environmental factors) that impede socialization. This is a set of relations sufficient to link elements of the patient’s clinical picture with the corresponding ICF code (with the severity determinant) and with the rehabilitation goals.

The information corresponds to the elements of the structure. The ICF code is a diagnosable disorder that is coded by the ICF classifier (see the ICF Handbook in Figure 1) and has a corresponding qualifier. The symptom complex is designed to group clinically significant observations, including test results. An observation is a unit that reflects clinically significant patient data (see the Observations Handbook in Figure 1). Each observation can be expressed both in a simple version of its description and using many elements or characteristics. Test results can be expressed in terms of both overall scores and scores on individual questions. Observation type represents membership of a category such as metric, heredity, lifestyle, etc.

**Figure 3:**
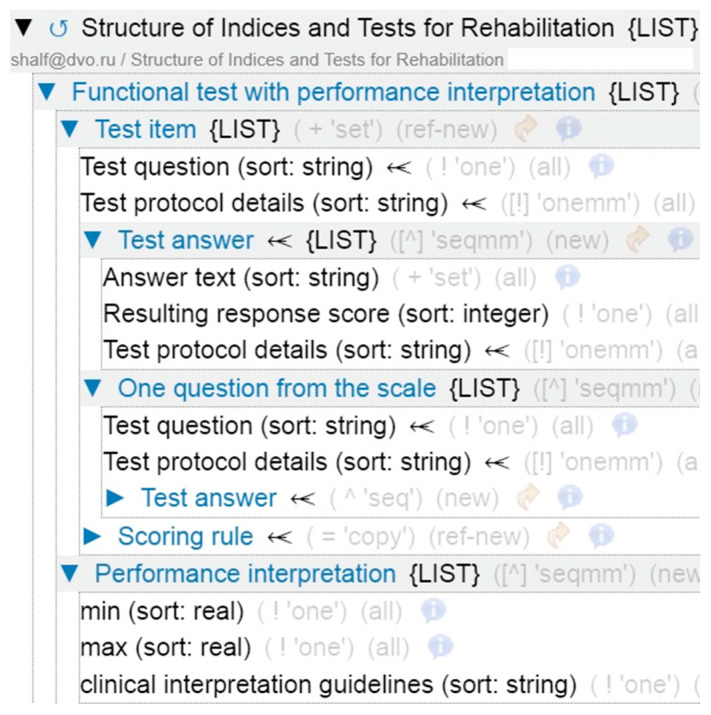
A fragment of the structure of tests and scales (clinimetric semantic model).

The resource for the rules of formal description of scale tests with interpretation has been formed (see Figure 3). It reflects the structure allowing a test to be presented with all possible questions, answer keys, and interpretation of results. In the process of analysis of the structure of the tests used to diagnose the patient’s rehabilitation ability, the following key vertices were identified: item, question, answer, calculation rule. The grouping of questions with the definition of question-answer pairs and the value of points corresponding to the answer is provided.

To support the entry of clinical data from patient examinations, surveys, and interviews (for rehabilitation purposes), a reference book of observations has been created on the medical portal of the IACPaaS platform. This book groups lists of different types of observations, described in a “simple” format or using multiple component characteristics. Any observation or fact of interest from medical history is provided. In order to optimize the access to the codes of disorders (for the diagnostic process), an image of the ICF classifier has been formed.

A precedent structure has been defined for situations in which reviewing a document on previous decisions made in similar circumstances is useful when prescribing a treatment regimen. This structure corresponds to that of a rehabilitation card. Precedents are archived based on this structure. To ensure conclusions are based on precedents, some precedents (i.e., previous decisions) are used directly or adapted to specific circumstances if necessary.

The structure of the rehabilitation medical records allows each specialist of the MDRT to have their own space for conducting collegial examinations and keeping diaries of daily examinations. Data is entered into the medical record in a formalized form: each section of the medical record is structured for the corresponding class of data used in medical practice.

### 3.1. The Method for Creating Decision Support Service Components

The IACPaaS technology for developing software systems with knowledge bases, as artificial intelligence technologies, includes digital tools for assembling software and information components into a unified system [14]. This assembly is based on a single set of semantic models for all generated and processed information in this domain. Using existing technology ontologies and knowledge graphs, a rehabilitation specialist support service was implemented. In addition to domain-oriented structures for knowledge and documents, and the nomenclature of terms used by specialists, structures were defined for declarative components that provide flexibility and scalability for the software “part” of the DSS, such as the user interface (GUI).

For the cooperation of the members of the multidisciplinary rehabilitation team, scenarios for the alternate examination of patients and the creation of a single rehabilitation record are provided. The resource for describing these scenarios contains a set of tabs (customizable by the engineer) with interface elements needed by specialists, reflecting their roles in the MDRT and in the rehabilitation process.

Since the chosen approach and the IACPaaS technology do not make software components dependent on the content of knowledge bases but on semantic models [14], it allows us to modify knowledge without changing the program code.

Modification of knowledge can be represented by replacement of previous blocks, clarification of some fragments, addition (often for new rehabilitation methods and programs) and extension with knowledge blocks (often for the following diseases and their stages not yet covered by automation).

Mechanisms for planned expansion and clarification of the knowledge base are provided. Specialists in the field and experts prepare an update for the knowledge. Then they transfer the update to a copy of the knowledge base in operation—the cloud resource of the platform. Currently, software converters are being created to translate knowledge from documents selected by experts into structured knowledge base modules.

After quality assessment and testing of the knowledge bases on control test cases, the updated resources replace the previously implemented bases (components) of the decision support system (virtual assistant).

### 3.2. Testing and Implementation

**Figure 4:**
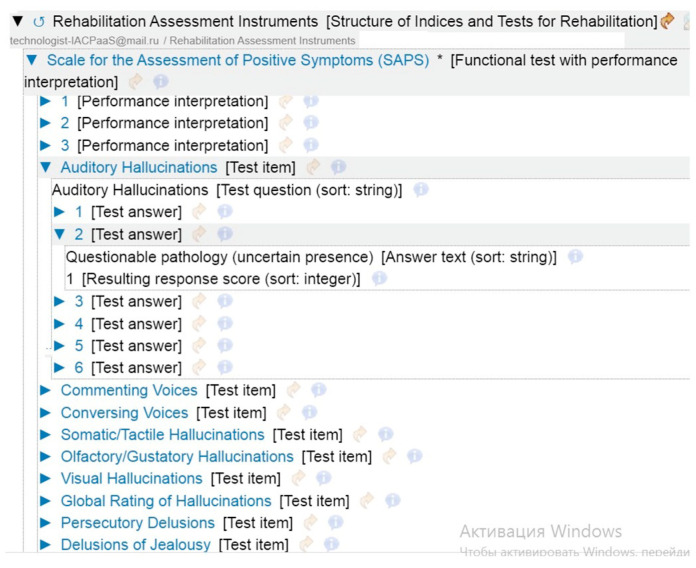
The fragment of resource with tests and surveys and scales.

Based on the proposed structure, the necessary knowledge bases were formed. The knowledge to create a specific ICF code based on clinical data is represented in a format familiar to rehabilitation specialists. Each test or scale has a strict order of the parameters to be examined and data on the interpretation of the results obtained (see Figure 4).

Intelligent support for the diagnostic phase, issuing the rehabilitation plan, and assigning the action plan are organized. A separate workspace is created for each member of the MDRT, taking into account the professional features of patient management. Each member of the team can conduct a survey, examination, appropriate tests with objective measurement scales, determine the goals of rehabilitation and prepare an individual plan for the patient. All conclusions of team members are available for expert evaluation and participate in the formation of the final protocol of results of work with the patient.

#### Clinical Validity

Clinical validity involves verifying the accuracy of the knowledge bases (and other information resources) and ensuring they are used correctly.

The requirements for the quality of knowledge bases may differ depending on the type of knowledge (explicit or implicit) and its source (expert, book, article, or document), among other factors. For instance, a knowledge base may be created by a team of experts or through the automated digitization of a text document.

From a practical point of view, the most significant aspect is the correctness of problem-solving using knowledge.

Clinical validity then refers to the correctness of decisions in relation to a set of reference situations (precedents). The reference database includes depersonalized medical records documenting the medical history of the disease of interest at the “cured” or “discharged” stage. These records include codes of violations, rehabilitation goals, and treatment measures, as well as their results.

In the process of assessing clinical validity, cases (i.e., results of assessing the work of the decision support service) of the following types are distinguished, in addition to the exact match of the result with the standard:

• inaccuracy-A: The system proposed an inaccurate solution; the treatment method is indicated, but the means and regimen are not proposed.
• inaccuracy-B: The system proposed other methods in addition to the ‘necessary’ treatment method.
• incompleteness-A:
  ○ not all “necessary” violation codes are offered;
  ○ not all goals are offered;
  ○ not all treatment measures are offered; (although the necessary ones are listed in the knowledge base).
• incompleteness-B: The system did not offer the correct solution due to the lack of knowledge about it in the system (no rules for determining codes, no such goals, no such methods).
• incorrect solution: Among alternatives there was not a single “necessary” part of decision:
  ○ violation code;
  ○ goal;
  ○ treatment measure.

## 4. Discussion

The telemedicine and decision support systems market is flooded with solutions. However, most of these solutions are based on the International Classification of Diseases (ICD). Our “rehabilitation solution” stands out because it is one of the first attempts at creating holistic software that focuses on the decision-making process rather than on clinical diagnoses. Instead, it is based on the functional status of the patient. Rehabilitation specialists use the ICF as the conceptual basis.

Furthermore, none of the known DSSs integrate such diverse types of knowledge necessary for this approach into a single semantic model: expert knowledge of rehabilitation specialists, formalized clinical guidelines, and precedent knowledge from real-world cases.

This approach’s key uniqueness lies in how it integrates knowledge to support a council of doctors’ decision-making process. Each member of the rehabilitation team views the patient through their own professional lens based on the ICF. The model of scenarios of joint work among doctors (MDRT Administration Format in Figure 1), who have a common goal but different perspectives on the patient, has made it possible to automate and enhance the consultation process.

In summary, our contribution is not just another “therapeutic application,” but rather, a conceptual framework that integrates knowledge and enables management of complex rehabilitation processes based on the ICF.

## 5. Conclusions

A set of interconnected semantic models is presented, which form the basis of the developed intelligent system of medical decision support for rehabilitation of patients. The IACPaaS cloud platform is used to implement this complex. The target resources created on its basis are the basic elements of the developed DSS for solving current rehabilitation problems.

At present, using the developed complex of ontologies, the system “Rehabilitation Councilium” for making an ICF diagnosis and defining rehabilitation aims has been implemented. Clinical trials of the proposed solution are being conducted.

The potential advantage of such systems is the combination of the most relevant knowledge with a high degree of personalization in rehabilitation planning. The personalization of the rehabilitation process is based on the consideration of detailed ICF-diagnosis and arbitrary set of individual patient characteristics, and observations of various state parameters in dynamics, accumulated in a formalized electronic health record about patients. The personalization of rehabilitation plans is embedded in the semantic template of the knowledge base itself: the relationships between concepts are not binary; a treatment measure depends on one or more identified dysfunctions and their severity, the characteristics of the body, and other factors, as well as the set goal and selected tasks.

The relevance of knowledge is based on the implemented procedure for regular updating of knowledge bases from trustworthy sources. The mechanisms of knowledge base expansion allow the system to easily adapt to new results of medical research.

The proposed ontological approach, combined with the cloud cover of the IACPaaS platform, makes it possible for groups of specialists of different competencies to participate independently in the development, including experts who provide and evaluate knowledge of the problem.

Currently, clinical information about patients entered via the GUI is saved in the electronic medical record (medical history) in the IACPaaS portal format and exported to JSON. Integration with medical information systems (MIS) is also planned via medical records in XML format. Currently, the knowledge and test base is filled partly from theory (based on literary sources) and partly from practice (information from practicing rehabilitation specialists in the field of traumatology and diseases of the central nervous system). There are two options for the base filling: manual editing or automatic translation of text into a structured resource.

The next stage is to expand knowledge related to assessing rehabilitation potential, predicting recovery; it is planned to extend the functionality to create an individual rehabilitation plan, as well as to study the scalability of the proposed solution to other diseases requiring rehabilitation.

Currently, the test and knowledge base have limited content. If psychologists, psychotherapists, and occupational therapists request it, the content will be supplemented, or the bases will be replaced. When choosing to automatically translate the provided texts into the bases, it is likely that additional training of the LLM on these specialists’ text materials will be required. Translation and adaptation into other languages (starting with English) is planned. In general, automatic translation mechanisms are available on the IACPaaS portal.

## Data Availability

Data supporting the presented results, including links to publicly available archived datasets analyzed or created during the study, are stored on the platform IACPaaS and are available upon request to the shalf@dvo.ru address in the form of json-files and screenshots or by sharing in the personal account of the registered user of IACPaaS.
